# A Novel Measurement Method of Mechanical Properties for Individual Layers in Multilayered Thin Films

**DOI:** 10.3390/mi10100669

**Published:** 2019-10-02

**Authors:** Zai-Fa Zhou, Mu-Zi Meng, Chao Sun, Qing-An Huang

**Affiliations:** Key Laboratory of MEMS of the Ministry of Education, Southeast University, Nanjing 210096, China; 220151259@seu.edu.cn (M.-Z.M.); sun_chaos@seu.edu.cn (C.S.); hqa@seu.edu.cn (Q.-A.H.)

**Keywords:** multilayered beam, Young’s modulus, residual stress, resonant frequency

## Abstract

Various multilayered thin films are extensively used as the basic component of some micro-electro-mechanical systems, requiring an efficient measurement method for material parameters, such as Young’s modulus, residual stress, etc. This paper developed a novel measurement method to extract the Young’s moduli and residual stresses for individual layers in multilayered thin films, based on the first resonance frequency measurements of both cantilever beams and doubly-clamped beams. The fabrication process of the test structure, the corresponding modeling and the material parameter extraction process are introduced. To verify this method, the test structures with gold/polysilicon bilayer beams are fabricated and tested. The obtained Young’s moduli of polysilicon films are from 151.38 GPa to 154.93 GPa, and the obtained Young’s moduli of gold films are from 70.72 GPa to 75.34 GPa. The obtained residual stresses of polysilicon films are from −14.86 MPa to −13.11 MPa (compressive stress), and the obtained residual stresses of gold films are from 16.27 to 23.95 MPa (tensile stress). The extracted parameters are within the reasonable ranges, compared with the available results or the results obtained by other test methods.

## 1. Introduction

Various multilayered thin films are extensively used as the basic component of some micro-electro-mechanical systems (MEMS), such as gas sensors [1], radio frequency (RF) components [2], micromachined mirrors [3], etc. Material mechanical parameters, such as Young’s modulus, residual stress, and so on, not only have great effect on the functions of MEMS devices, but also have great influence on yield, service life, and the working reliability of MEMS devices. However, compared with bulk material, the major difficulties encountered during measuring the mechanical properties of thin films are due to the small dimensions of the sample, which are not amenable to testing by conventional means. Furthermore, most of current measuring methods for mechanical parameters of thin film materials, such as nanoindentation [4], uniaxial tensile testing of free-standing film [5], beam bending [6], bulge tests [7], etc. are suitable for single-layer thin film materials, and some of them are not beyond to in situ methods. In situ measurement makes it possible to extract and analyze the mechanical parameters together with the device fabrication processes. This is significantly helpful, considering the mechanical parameters in the micromachined film depends on not only the process conditions but also the fabrication processes. Therefore, an in situ extraction method of material properties for multilayered films is expected [8,9,10,11,12]. During the past several years, some methods have been presented for extracting mechanical parameters [10,11,12]. The method based on the resonant frequency of doubly-clamped multilayered beams is relatively complex and the inaccuracy from the iterative computation is a little high, because four-element nonlinear equations need to be solved [10,11]. The most serious problem is that inappropriate setting of initial values will lead to divergence of the iteration process of solving the equations, which makes it impossible to obtain the correct parameter values. This significantly limits its practicability. An approach using multilayered cantilever beams has been presented based on the first resonance frequency measurement, but only the Young’s modulus of each layer for multilayered films can be extracted [12]. 

In this paper, a novel method is proposed to directly measure both the Young’s moduli and residual stresses for the multilayered thin films simultaneously. The Young’s moduli and residual stresses can be extracted by measuring first the resonant frequencies of multilayered cantilever beams and doubly-clamped beams with different widths for each layer, together with the deflections of multilayered beams. The validity of this approach has been studied using finite element analysis (FEA) tools. As a multilayered example, test structures for gold/polysilicon bilayer beams are fabricated and tested. A digital holographic microscopy DHM R2200 (Lyncée Tech, Lausanne, Switzerland) is adopted to determine whether the beams have deflection, and to obtain the values of the curvature radius of the cantilever beams. A scanning laser Doppler vibrometer system (Polytech GmbH, Berlin, Germany) is adopted to measure the first resonant frequency of the multilayered cantilever beams and doubly-clamped beams. The obtained Young’s moduli of polysilicon films are from 151.38 GPa to 154.93 GPa, and the obtained Young’s moduli of gold films are from 70.72 GPa to 75.34 GPa. The obtained residual stresses of polysilicon films are from −14.86 to −13.11 MPa, and the obtained residual stresses of gold films are from 16.27 to 23.95 MPa. The obtained Young’s moduli are found to be in agreement with the values obtained by nanoindentation method. The obtained residual stress values are found to be in agreement with the run data values from the fabrication factory. The extracted parameters are all within reasonable ranges, compared with the available results.

## 2. Theory of the Test Structure 

### 2.1. Basic Theory of the Multilayered Beam

**Figure 1:**
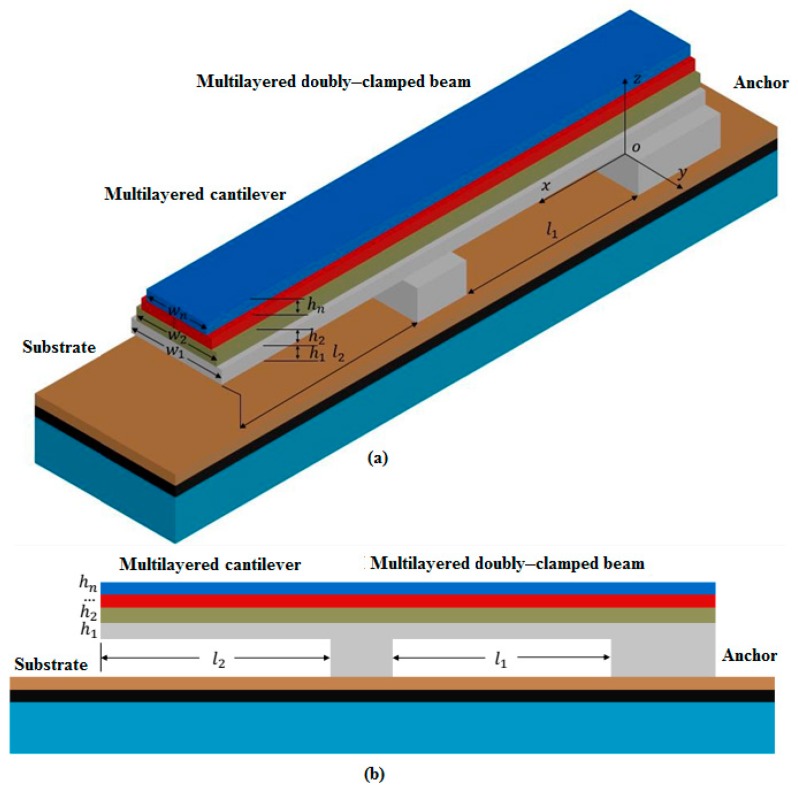
Schematic diagram of a test structure with a cantilever beam and a doubly-clamped beam. (**a**) 3D view; (**b**) Side view.

The approach is to extract the Young’s moduli and residual stresses for each layer in multilayered films, based on the test structure including *n*-layer cantilever beams and *n*-layer doubly-clamped beams with different widths for each layer, as shown in Figure 1. These beams can also be designed to be structurally independent of each other, as single *n*-layer cantilever beams and single *n*-layer doubly-clamped beams. The length of the doubly-clamped beam is *l*_1_ and the length of the cantilever beam is *l_2_*. The width, thickness, Young’s modulus, residual stress, Poisson’s ratio, and density of the *i*th layer are *w_i_*, *h_i_*, *E_i_*, σ_i_, *ν_i_*, and *ρ_i_*, respectively. During the following analysis, a positive stress represents tensile stress and a minus stress represents compressive stress. The length, width, and height direction of the beam are along the *x*, *y* and *z* axes, respectively.

For multilayered beams studied in the paper, the beam width is five times greater than the beam thickness, so the beam can be considered to be wide. Thus, the Young’s modulus *E_i_* should be substituted by the effective Young’s modulus ${\tilde{E}}_{i}=\frac{E_{i}}{1-v_{i}{}^{2}}$. As shown in Figure 1, the height direction of the beam is assumed to be along the *z* axis, and the position of the beam bottom is z*_0_* = 0, while the position of the top for the *i*th layer is $z_{i}=\sum_{j=1}^{i}h_{i},(i,j=1,2,\ldots ,n)$. The distance from the neutral axis to the bottom of the beam is defined as *z_c_*, which can be given by [13]

(1) $$z_{c}=\frac{\sum_{i=1}^{n}{\tilde{E}}_{i}w_{i}\left(z_{i}^{2}-z_{i-1}^{2}\right)}{2\sum_{i=1}^{n}{\tilde{E}}_{i}w_{i}\left(z_{i}-z_{i-1}\right)}$$

The moment of inertia of the *i*th layer, *I_i_*, with respect to the neutral axis of the beam can be expressed by

(2) $$I_{i}=\int_{i}{\left(z-z_{c}\right)}^{2}dA_{i}=\frac{1}{3}w_{i}\left[{\left(z_{i}-z_{c}\right)}^{3}-{\left(z_{i-1}-z_{c}\right)}^{3}\right],(i,j=1,2,\ldots ,n)$$

where *A_i_* is the cross-sectional area of the *i*th layer. For a multilayered beam, the bending stiffness $\bar{EI}$, the axial stiffness $\bar{EA}$, the linear density $\bar{\rho A}$ and the axial load $\bar{\sigma A}$, are defined by the sum of the individual terms for each layer in the beam as given by the following equations [14]:

(3) $$\bar{EI}=\sum_{i=1}^{n}{\tilde{E}}_{i}I_{i}$$

(4) $$\bar{EA}=\sum_{i=1}^{n}{\tilde{E}}_{i}A_{i}$$

(5) $$\bar{\rho A}=\sum_{i=1}^{n}\rho_{i}A_{i}$$

(6) $$\bar{\sigma A}=\sum_{i=1}^{n}{\tilde{\sigma }}_{i}^{0}A_{i}$$

### 2.2. The Resonant Frequency of n-Layer Doubly-clamped Beam 

According to the theory of beams [11,15,16], residual stress can be regarded as an axial load, and the axial load has an important influence on the mechanical properties of the doubly-clamped beam. When the residual stress is tensile stress, the effective transverse elastic coefficient of the beam will be increased. Thus, it is more difficult for transverse bending to occur, and there will be no initial deformation. On the contrary, when the residual stress is compressive stress, the situation will be more complicated. The effective transverse elastic coefficient of the beam will be reduced correspondingly. Transverse bending then occurs more easily and the doubly-clamped beam could even be buckled.

An *n*th critical buckling load *P*^(n)^ is defined as the axial load sufficient to maintain the doubly-clamped beam in slightly bent form. In this paper, *P*^(n)^ is a compressive stress and a negative value. If the load *P* is greater than the critical buckling load value *P*^(n)^ (P > P^(n)^,|P| < |P^(n)^|), the doubly-clamped beam will remain straight. If *P* < *P*^(n)^ (|*P*| > |*P*^(*n*)^| ), the doubly-clamped beam will be initially deflected and a postbuckling displacement *b*$\phi_{n}$(*x*) is induced, where *b* is the dimensional scaling constant and $\phi_{n}$(*x*) is the *n*th buckling mode shape. In other words, if a lateral force is applied then a small deflection will be produced. However, the deflection will vanish, and the doubly-clamped beam will restore to its initial form when the lateral force is removed. On the contrary, if *P* is gradually reduced (compressive stress is a negative value), a condition will be reached in which the straight form of equilibrium becomes unstable and a small lateral force will cause a deflection that will not vanish when the lateral force is removed. It should be pointed out that when the initial deformation is small, the behavior equation for the flat state can be used to approximate it with small error. This is the case which will be discussed below.

For an unbuckled *n*-layer doubly-clamped beam, the axial load *P* = $\bar{\sigma A}$ is greater than the critical value *P*^(n)^, so the beam remains flat. If the unbuckled doubly-clamped beam is with a uniform cross section along its length, the natural frequencies ω for this beam can be included in the eigenvalue equation [15,16]:

(7) $$F_{U}=\left|U\right|=\left|\begin{array}{cccc} 0 & 1 & 0 & 1\\ \lambda_{1} & 0 & \lambda_{2} & 0\\ \sin\left(\lambda_{1}l_{1}\right) & \cos\left(\lambda_{1}l_{1}\right) & \sinh\left(\lambda_{2}l_{1}\right) & \cosh\left(\lambda_{2}l_{1}\right)\\ \lambda_{1}\cos\left(\lambda_{1}l_{1}\right) & -\lambda_{1}\sin\left(\lambda_{1}l_{1}\right) & \lambda_{2}\cosh\left(\lambda_{2}l_{1}\right) & \lambda_{2}\sinh\left(\lambda_{2}l_{1}\right) \end{array}\right|=0$$

where $\lambda_{1}=\sqrt{\left(\sqrt{\beta_{1}^{2}+4\beta_{2}}-\beta_{1}\right)/2}$,$\lambda_{2}=\sqrt{\left(\sqrt{\beta_{1}^{2}+4\beta_{2}}+\beta_{1}\right)/2}$,$\beta_{1}=\frac{\bar{\sigma A}}{\bar{EI}}$,$\beta_{2}=\frac{\bar{\rho A}}{\bar{EI}}\omega^{2}$. Equation (7) has numerous solutions ω*_n_*(*n* = 1, 2, 3, … *i*, …), corresponding to the *i*th resonant frequencies of the resonant beam. Theoretically, Equation (7) belongs to transcendental equation, and there is no analytical solution. Proper numerical method should be carefully implemented to obtain corresponding numerical solutions.

To determine the deformation of the doubly-clamped beam, a DHM R2200 (Lyncée Tech., Lausanne, Switzerland) is used. The DHM can produce an off-axis hologram through the interference between the wave reflected by the sample and a reference wave. The profiles of the beams extracted from the DHM measurement can be depicted. From the depicted figures, it can be observed that some beams are buckled, and others are slightly bent or even flat. 

For n-layer doubly-clamped films, there are many combinations of residual stresses in each layer of material. However, the goal of this paper is to extract material parameters, so it is necessary to simplify the testing structure and extraction method as much as possible. Therefore, this paper mainly studies the extraction method without significant buckling after release. The extension of the application scope of this method is realized by designing reasonable structure with proper size. For a doubly-clamped beam with length less than 200 μm, if its maximum displacement at the center is less than 1.5% of the total length of the beam, it can still be considered as slightly bending. For example, when the residual stress in the bottom layer is −75 MPa and the residual stress is –70 MPa in the top layer for two-layer cases, the maximum displacement at the center of a doubly-clamped beam with 120 μm length and 11μm width is less than 0.02 μm; thus, it can be considered as slightly bending. Obviously, if the stress of one layer is compressive and the stress of the other layer is tensile stress, the effective testing range of this method will be wider. More detailed explanations will be provided in the latter parts of the paper.

### 2.3. The Resonant Frequency of Cantilever Beam

For unbuckled *n*-layer cantilevers after release, the approximate analytic formula of its *i*th resonant frequencies can be obtained by [17]

(8) $$f_{i}=\frac{1}{2\pi }{\left(k_{i}l_{2}\right)}^{2}\sqrt{\frac{\bar{EI}}{\bar{\rho A}l_{2}{}^{4}}}$$

where *k_i_l*_2_ satisfies the function *cos*(*k_i_l*_2_)*cosh*(*k_i_l*_2_) = −1; thus, *k_1_l_2_* = 1.875, *k_2_l_2_* = 4.694, *k_3_l_2_* = 7.855, …. So, the approximate analytic formula of the first resonant frequency for an unbuckled n-layer cantilever beam after release can be expressed as

(9) $$f_{1ucf}=\frac{1.875^{2}}{2\pi l_{2}{}^{2}}\sqrt{\frac{\bar{EI}}{\bar{\rho A}}}$$

where subscript 1 indicates the first resonant frequency, *u* indicates that the cantilever beam is unbuckled, and *cf* indicates that the boundary condition is cantilever.

Due to the unmatched thermal stress, or for other reasons, in most cases n-layer cantilever beams will be buckled after release. When the cantilever beam is buckled, the influence of deflection to resonance frequency should be considered. Assuming that the curvature radius of a cantilever beam after release is *R*, a function describing its first resonant frequency can be obtained as follows [11,16,17]:

(10) $$\frac{1}{\sqrt{\chi_{n}^{2}-1}}\sin\left(\sqrt{\chi_{n}+1}\frac{l_{2}}{R}\right)\sinh\left(\sqrt{\chi_{n}-1}\frac{l_{2}}{R}\right)+\cos\left(\sqrt{\chi_{n}+1}\frac{l_{2}}{R}\right)\cosh\left(\sqrt{\chi_{n}-1}\frac{l_{2}}{R}\right)+1=0$$

where

(11) $$\chi_{n}^{2}=\bar{\rho A}R^{4}{\left(2\pi f_{n}\right)}^{2}/\bar{EI}$$

**Figure 2:**
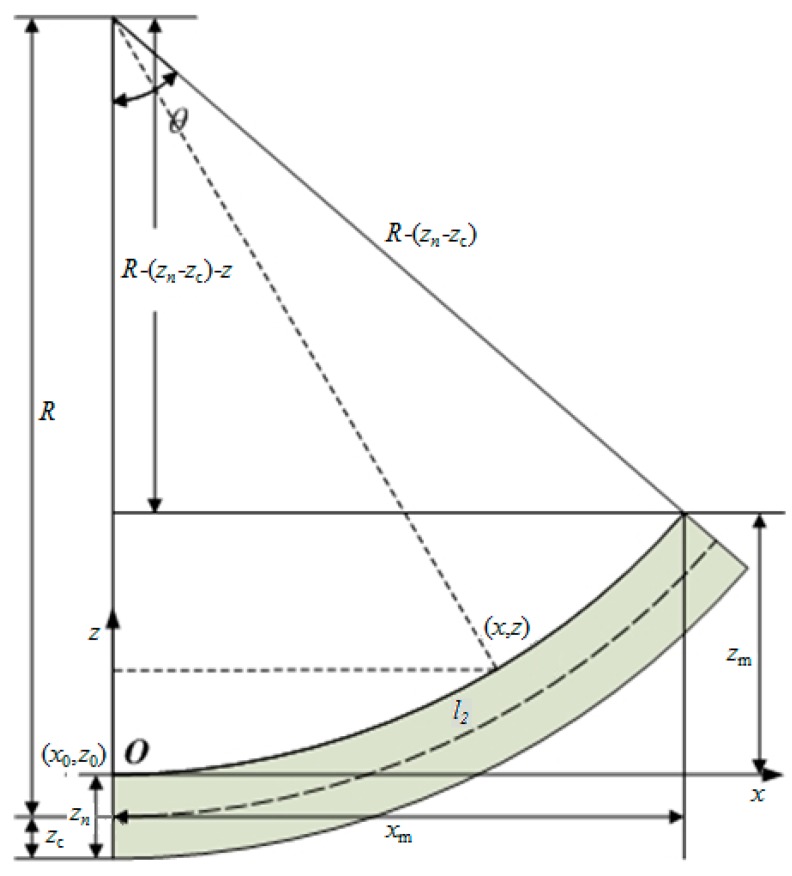
Schematic diagram of cantilever beam with deflection radius of *R* after deflection.

Assuming that the cantilever beam has a uniform curvature radius, *z_m_* is the maximum deflection of cantilever beam, *l_2_* is the length of the unbuckled cantilever beam, a simplified equation can be obtained to fit out its curvature radius $R=l_{2}{}^{2}/(2z_{m})$ [12]. This is suitable for obtaining the curvature radius *R* from FEA results. In order to improve the calculation accuracy for practical tests using some equipment, such as DHM R2200, which will determine the curvatures of the cantilever beam from the contour curve profiles of the cantilever beam top surface, a novel model is presented in this paper. As shown in Figure 2, *x_m_* is the length of buckled cantilever, *z*_n_ is the thickness of the multilayer cantilever and *z_c_* is the height of the neutral axis. Assuming the coordinates of certain points on the contour curve of the beam are (*x*_0_,*z*_0_), and the coordinates of any other point on the contour curve are (x,z), then, according to the Pythagorean theorem, we can get (*x − x*_0_)^2^ + [*R −* (*z_n_ − z*_c_) *−* (*z − z_0_)*]^2^ = [*R −* (*z_n_ − z*_c_)]^2^. Considering that *z_n_* is much smaller than the curvature radius of the cantilever beam, then we have (*z − z*_0_)^2^ *−* 2*R*(*z − z*_0_) + (*x − x*_0_)^2^ = 0. If *x* is regarded as a known number and *z* is regarded as an unknown number, the contour curve equation of the cantilever beam top surface can finally obtained as follows:

(12) $$z=R-\sqrt{R^{2}-{\left(x-x_{0}\right)}^{2}}+z_{0}$$

The curvature radius *R* of the deflection cantilever beam can then be quickly obtained by nonlinear fitting of the measured profile curve of the top surface according to Equation (12).

Equation (10) is also a transcendental equation, and this kind of equation can only be solved approximately using numerical methods. There are many approximate methods to solve the transcendental equation, such as Newton’s method and Mueller’s method, among others [18,19]. There are numerous sets of solutions for Equation (10), $\chi_{n}$(*n* = 1, 2, 3,…*i*,…). Each $\chi_{n}$ has the corresponding *i*th resonance frequency. However, to ensure that the first resonance frequency is obtained, the solution of Equation (10) should be the smallest one, $\chi_{1}$. By applying the smallest value $\chi_{1}$ into Equation (10), we can get the changed form of Equation (10) as follows:

(13) $$f_{1bcf}=\frac{1}{2\pi }\cdot {\left(\frac{\chi_{1}^{2}\cdot \bar{EI}}{\bar{\rho A}\cdot R^{4}}\right)}^{1/2}$$

where *b* indicates that the cantilever is buckled.

It should be noted that if the curvature radius of the cantilever beam tends to infinity, then we get the same result as Equation (8), indicating that the vibrations of both the buckled and unbuckled multilayered cantilever beams have little difference if the curvature radius of the cantilever beams tend to infinity.

### 2.4. The Extraction Approaches of Material Parameters

Based on the above analysis and discussion, this paper presents a novel method for extracting the mechanical parameters of multilayered thin films according to the test structure as shown in Figure 1. 

According to Equations (8) and (13), it only needs *n* types of cantilevers to obtain Young’s moduli for each layer for an *n*-layer films. Assuming the widths of each layer from the bottom to the top for the *j*th cantilever are *w_j_*_1_, *w_j_*_2_, …, *w_jn_*, (1 ≤ *j* ≤ n) and the widths of each layer from the bottom to the top for the *k*th cantilever are *w_k_*_1_, *w_k_*_2_, …, *w_kn,_* (1 ≤ *k* ≤ *n*, *k* ≠ *j*), if the beams are designed to have the same length, the vector (*w_j_*_1_, *w_j_*_2_, …, *w_jn_*) and the vector (*w_k_*_1_, *w_k_*_2_, …, *w_kn_*) should be designed to be linearly independent [11]. Since the first layer of all cantilever beams has the same width, it is only necessary to ensure that the width combinations from the second layer to the top layer for each cantilever beam are not exactly the same. Assuming that the measured first resonance frequencies of the *n* types of cantilevers are *f*_1_, *f_2_*, …, *f_n_*, a set of equations can be deduced as given by

(14) $$\left\{ \begin{array}{l} f_{1xcf,1}({\tilde{E}}_{1},{\tilde{E}}_{2},\ldots ,{\tilde{E}}_{n},w_{12},w_{13},\ldots ,w_{1n},l_{2})-f_{1}=0\\ f_{1xcf,2}({\tilde{E}}_{1},{\tilde{E}}_{2},\ldots ,{\tilde{E}}_{n},w_{22},w_{23},\ldots ,w_{1n},l_{2})-f_{2}=0\\ \ldots \\ f_{1xcf,i}({\tilde{E}}_{1},{\tilde{E}}_{2},\ldots ,{\tilde{E}}_{n},w_{i2},w_{i3},\ldots ,w_{in},l_{2})-f_{i}=0\\ \ldots \\ f_{1xcf,n}({\tilde{E}}_{1},{\tilde{E}}_{2},\ldots ,{\tilde{E}}_{n},w_{n2},w_{n3},\ldots ,w_{nn},l_{2})-f_{n}=0 \end{array}\right.$$

where ${\tilde{E}}_{i}$ is the effective Young’s modulus of the *i*th layer material, x=b when the cantilever is buckled, and x=u when the cantilever is unbuckled. DHM R2200 was used to distinguish the buckled from the unbuckled, and also to measure the curvature radius of cantilever after release. 

It should be mentioned that effective Young’s modulus ${\tilde{E}}_{j}$ can be obtained from Equation (14) rather than Young’s modulus *E_i_* because the Poisson’s ratio *ν_i_* in each layer is unknown. To make results more intuitive, the paper sets exact values to the Poisson’s ratio in each layer. The way to extract the Poisson’ ratio *ν_i_* is introduced in [20,21,22]. The Poisson’s ratio of each layer in a certain rational range has little effect on the results of the Young’s modulus [12]. For example, the rational range of the Poisson’s ratio in the polysilicon layer is 0.2–0.25 and the rational range of the Poisson’s ratio in the gold layer is 0.38–0.45. This paper assumes 0.22 and 0.42 as the Poisson’s ratios in the polysilicon layer and gold layer, respectively. It is obvious that results obtained based on this assumption have little differences among the results obtained based on other values in the rational range.

According to Equation (7), there should be at least *n* types of doubly-clamped beams to obtain the residual stresses for each layer, if the Young’s moduli for each layer have been obtained according to Equation (13). The widths of the *j*th doubly-clamped beam are defined as *w’_j_*_1_, *w’_j_*_2_, …, *w’_jn_* and the widths of the *k*th doubly-clamped beam are defined as *w’_k_*_1_, *w’_k_*_2_, …, *w’_kn_*. Similarly, if the beams are designed to have the same length, the vector (*w’_j_*_1_, *w’_j_*_2_, …, *w’_jn_*) and the vector (*w’_k_*_1_, *w’_k_*_2_, …, *w’_kn_*) should be designed to be linearly independent. Since the first layer of all doubly-clamped beams has the same width, it is only necessary to ensure that the width combinations from the second layer to the top layer for each doubly-clamped beam are not exactly the same. Assuming that the measured first resonance frequencies of the *n* types of doubly-clamped beams are *f’*_1_, *f’_2_*, …, *f’_n_*, a set of equations can be deduced by

(15) $$\left\{ \begin{array}{l} F_{U,1}\left(2\pi f_{1}^{\prime },{\tilde{E}}_{1},{\tilde{E}}_{2},\ldots ,{\tilde{E}}_{n},\sigma_{1},\sigma_{2},\ldots ,\sigma_{n},w_{12}^{\prime },w_{13}^{\prime },\ldots ,w_{1n}^{\prime },l_{1}\right)=0\\ F_{U}{{}_{,}}_{2}\left(2\pi f_{2}^{\prime },{\tilde{E}}_{1},{\tilde{E}}_{2},\ldots ,{\tilde{E}}_{n},\sigma_{1},\sigma_{2},\ldots ,\sigma_{n},w_{22}^{\prime },w_{23}^{\prime },\ldots ,w_{2n}^{\prime },l_{1}\right)=0\\ \ldots \\ F_{U}{{}_{,}}_{i}\left(2\pi f_{i}^{\prime },{\tilde{E}}_{1},{\tilde{E}}_{2},\ldots ,{\tilde{E}}_{n},\sigma_{1},\sigma_{2},\ldots ,\sigma_{n},w_{i2}^{\prime },w_{i3}^{\prime },\ldots ,w_{in}^{\prime },l_{1}\right)=0\\ \ldots \\ F_{U,n}\left(2\pi f_{n}^{\prime },{\tilde{E}}_{1},{\tilde{E}}_{2},\ldots ,{\tilde{E}}_{n},\sigma_{1},\sigma_{2},\ldots ,\sigma_{n},w_{n2}^{\prime },w_{n3}^{\prime },\ldots ,w_{nn}^{\prime },l_{1}\right)=0 \end{array}\right.$$

where ${\tilde{E}}_{j}$ and $\sigma_{j}$ are the Young’s modulus and the residual stress of the *j*th layer material. 

Thus, the Young’s moduli for each layer are calculated by Equation (14), and then the residual stress of each layer can be solved by Equation (15). In this way, only the unknown parameters of residual stresses for each layer need to be solved by numerical iteration, which greatly simplifies the complexity of the solution. Namely, the selection of initial iteration values becomes less strict, and it becomes easier to guarantee the convergence of the iteration process.

## 3. Test Structure Design and Simulation Analysis

Theoretically speaking, the method presented in this paper can be used to extract the mechanical parameters of multilayered thin films. From the view of practical application, the extractions of mechanical parameters of three-layer films and two-layer films are its typical applications. The purpose of this paper is to prove the feasibility of our approaches, so test structures with two layers are fabricated using the PolyMUMPs process (MEMSCAP Inc., Durham, NC, USA) for simplification. The design, analysis and tests for the test structures of two layers can be extended to more complicated cases with more than two layers. 

**Figure 3:**
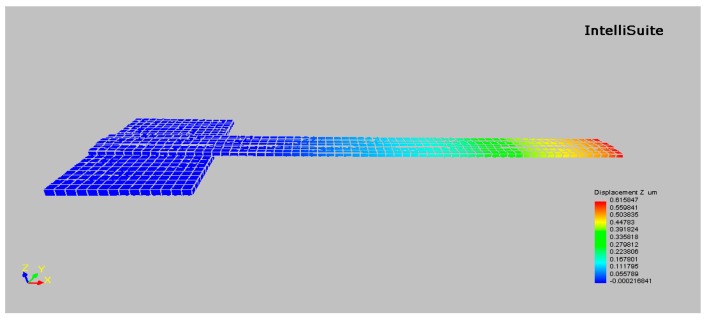
Solid model and analysis results of the Cantilever beam 4 in Intellisuite 8.9 software.

According to the design rules of PolyMUMPs process, several types of beams are designed for experimental verification, as listed in Table 1. Actually, before we determine the experimental scheme and the fabrication experiments, test structures for two layer films have been analyzed using a FEA tool, Intellisuite 8.9 software (Jiangsu Intellisense Technology Co., Ltd, Nanjing, China) [23]. Firstly, to avoid the situation that the bottom layer of the cantilever adheres to the substrate, test structures with suitable dimensions should be designed. For example, assuming the residual stress is +60 MPa in the bottom layer and the residual stress is –60 MPa in the top layer, the maximum downward deflection for the test structure of cantilever beam 6 is about 1.46 μm according to the simulations. Considering the gap between the Poly2 and the substrate for PolyMUMPs process can approach 2.75 μm, this may guarantee a relatively small possibility of substrate adhesion. For some other cases, the gap formed by releasing sacrificial layer between the beam and the substrate is smaller. Obviously, substrate adhesion may happen if all other conditions remain unchanged. However, the dimensions of the test structures can be accordingly adjusted based on the specific process in order to realize a wider application range. For example, if the residual stress values are still +60MPa in the bottom layer and −60 MPa in the top layer, the maximum downward deflection for the cantilever beam with new dimensions (90 μm length, 10 μm width for bottom layer and 6 μm width for top layer) is about 0.96 μm according to the simulations. Thus, if the gap between the cantilever beam and the substrate for other fabrication line is reduced to 2.00 μm, this may still guarantee a relatively small possibility of substrate adhesion. Secondly, in order to keep the doubly-clamped beam satisfied with slight bending, the test structures should also be properly designed. According to the simulations, assuming the residual stress is −75 MPa in the bottom layer and −70 MPa in the top layer, the maximum deflection after release for the test structure of Doubly-clamped beam 4 is less than 0.02 μm. Thus, this situation can still be considered as slightly bending. Finally, three groups of material parameters with different Young’s moduli and residual stresses for the simulation are listed in Table 2. The first natural resonant frequencies and deflection profiles of the test structures are obtained by the FEA simulations for Cantilever beam 1, Cantilever beam 2, Doubly-clamped beam 1 and Doubly-clamped beam 2 as listed in Table 1. Figure 3 shows the solid model of the cantilever beam 4 in Intellisuite 8.9 software. The analysis results are then put back into Equations (14) or (15) to calculate the Young’s moduli and residual stresses. Table 3 shows the comparison results between the material parameter values set to the FEA analysis and those calculated from FEA results. Table 3 indicates that the proposed approach is effective. This also verifies that above assumption related to the approximation of slight bending of a doubly-clamped beam is reasonable. The approximation only produces a small inaccuracy for the simulation results of Doubly-clamped beam 4.

We should acknowledge that the relative error in Table 3 is slightly high. We think there may be three main reasons for this. One reason is that there must be some errors between the results from the analytical equations and the results from finite element analysis. Thus, there will be errors when the results from finite element analysis are used to implement the iterative solution process of the analytical equations, especially when the beams are in a little bending (e.g., high compressive stress for both layers). The second reason is that the numerical solutions of Equation (15) need complicated iteration processes, and the currently available Newton iteration methods are still not accurate enough for this complex case. So, improvement of existing iteration methods has become an important research task in our future. Finally, the iteration errors are related to initial values. When we set input parameters with larger values (tensile), the relative error seems to be a little bit smaller. 

## 4. Experiments and Discussions

**Figure 4:**
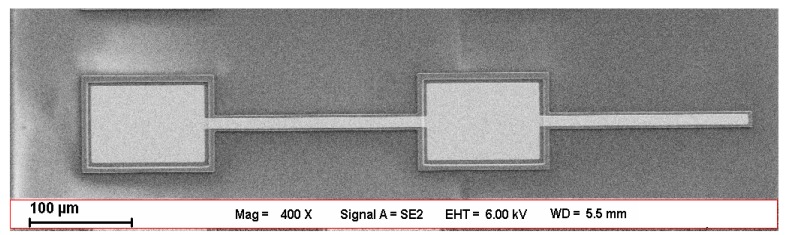
Photograph of the test structure with a cantilever beam and a doubly-clamped beam. The lengths of the cantilever beam and the doubly-clamped beam are 200 μm, the width of the polysilicon layer is 15 μm and the width of the gold layer is 9 μm.

PolyMUMPs is one of the three standard processes offered by MEMSCAP Inc., as part of their Multi-User MEMS Process (MUMPs) program. We plan to validate the Young’s moduli with the data of nanoindentation experiments, and to validate the residual stresses by using the residual stress values for PolyMUMPs directly provided by MEMSCAP Inc. This is one of the reasons why we choose PolyMUMPs standard process for experimental verification, because it is a convention for MEMSCAP Inc. to provide consumer the Run data with residual stresses after each Run. Another reason is that the excellent stability of PolyMUMPs process, so the uncertainties during our verification process can be notably reduced. Its basic fabrication steps are as follows. At first, a 600 nm silicon nitride layer is deposited on the wafers using LPCVD (Low Pressure Chemical Vapor Deposition). It acts as the insulation between the substrate and electrical surface layers. It is followed directly by the LPCVD deposition of a 500 nm polysilicon layer Poly0, which is the electrical layer for ground plane and is below the first mechanical layer. After the etching of Poly0, a 2.0 μm LPCVD PSG (Phosphosilicate glass) sacrificial layer is formed. It is the first sacrificial oxide layer, providing the gap between the substrate or silicon nitride and Poly1. The first polysilicon structural layer Poly1 is deposited by LPCVD as well with a thickness of 2.0 μm, and the second sacrificial oxide layer is formed on top of it, with a thickness of 0.75 μm, providing the gap between Poly1 and Poly2. The second structural layer Poly2 (1.5 μm thick) is deposited after the etching of the second oxide layer, followed by a 0.5 μm gold layer that provides for probing, bonding, electrical routing, etc. After the deposition and patterning of all seven layers comes the hydrofluoric acid release, which etches away the two oxide layers and forms the gaps. Figure 4 shows the fabrication result for the test structure with a cantilever beam and a doubly-clamped beam.

**Figure 5:**
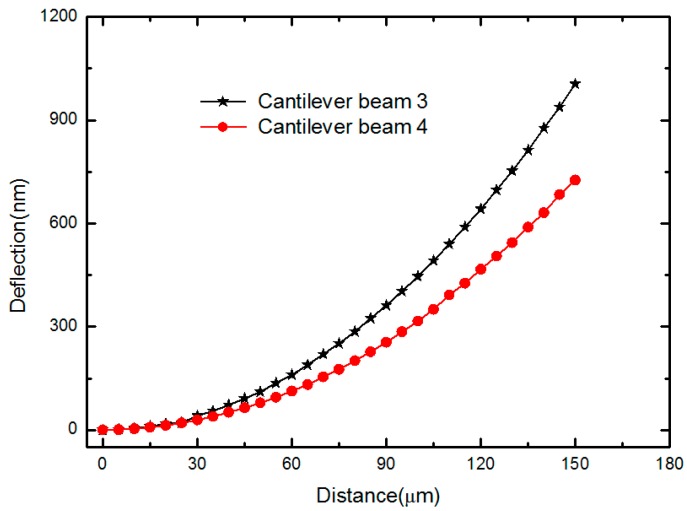
Deflection curve in the phase diagram for the Cantilever beam 3 and Cantilever beam 4 in DHM measurement.

**Figure 6:**
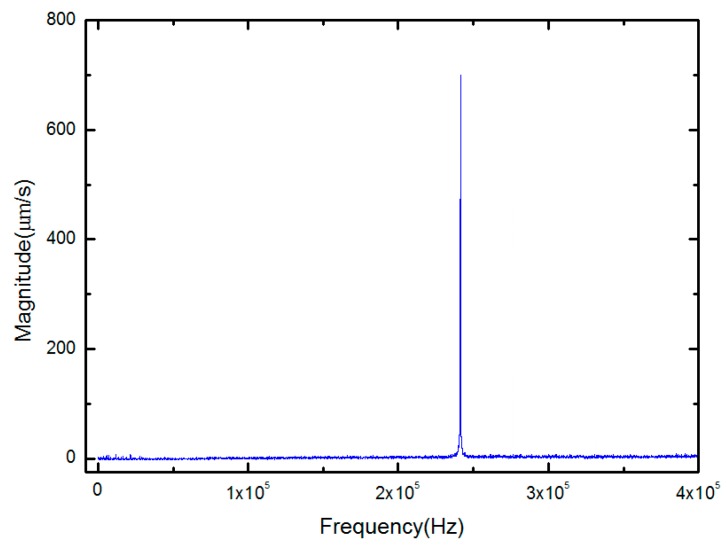
Frequency response curve of the Doubly-clamped beam 2.

The deflection curve from the DHM measurement for the Cantilever beam 4 and Cantilever beam 4 is depicted as shown in Figure 5. The maximum deformations of beam center for doubly-clamped beams with 200 μm length and 120 μm length were both less than 0.01 μm, and they should all be treated as slightly bending and unbuckled. The obtained curvature radii of the cantilever beams obtained by using Equation (12) are summarized in Table 4. After that, the first resonance frequency of the test structures was measured by using a scanning laser Doppler vibrometer system (Polytech GmbH, Berlin, Germany) with a hardware modulus of MSV-400-M2. The test structures were excited by piezoelectric plumbum zirconate titanate (PZT) ceramic slices with a periodical chip signal. By applying driven signal with frequencies ranging from 1 kHz to 1 MHz, and the resonant frequencies were determined from the frequency response functions [24]. Figure 6 shows the frequency response curve of the Doubly-clamped beam 2. The measured first frequencies for various beams of different test structures are listed in Table 4.

Assuming that the Poisson’ ratio and density of the polysilicon layer are 0.22 and 2330 kg/m^3^, respectively, and the Poisson’ ratio and density of the metal are 0.42 and 19,300 kg/m^3^, respectively, the effective Young’s moduli and residual stresses were extracted simultaneously by Equations (14) and (15). Six types of combination of test structures were as listed in Table 5. Each combination is corresponding to 2 groups of solvable equations from equations (14) and (15). The Young’s moduli and residual stresses of both layers are then extracted. For example, Cantilever beam1, Cantilever beam2, Doubly-clamped beam1 and Doubly-clamped beam2 can be combined to obtain the Young’s moduli of Poly2 and the Au layer, together with the residual stresses of Poly2 and the Au layer. However, Cantilever beam1, Cantilever beam2, Doubly-clamped beam3 and Doubly-clamped beam4 can also be combined to obtain the same parameters. Furthermore, it should be notice that Cantilever beam1 and Cantilever beam2 are combined to obtain the Young’s moduli of Poly2 and the Au layer. With these obtained Young’s Modulus values, the combination of Doubly-clamped beam1 and Doubly-clamped beam2, or the combination of Doubly-clamped beam1 and Doubly-clamped beam2 can be used to obtain the residual stresses of Poly2 and the Au layer.

**Figure 7:**
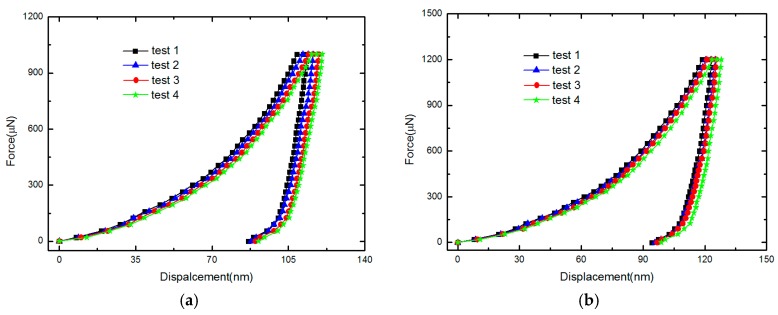
Force-displacement curves for a 500 nm thick Au layer for different maximum loads in TI-750 nanoindentation: (**a**) 1000 μN maximum load (**b**) 1200 μN maximum load.

Nanoindentation is a testing method that has been widely used to measure the elastic properties of micro- and nano-materials. With the feedback control system applied in this instrument, precise force-displacement data and curve can be obtained, from which hardness and elastic modulus can be determined [25]. To verify the obtained Young’s moduli of polysilicon and gold, nanoindentation method with the Berkovich diamond indenter using TI-750 nanoindentation (Hysitron, Inc., Minneapolis, MN, USA) has been implemented on the anchor regions of the test structures. The Au layer on some anchors has been specially etched away during the fabrication process, for the nanoindentation experiments for Poly2 layer. As the influence from the substrate will affect the test accuracy, we tried to restrict the penetration depth to less than 140 nm for the Au layer and 250 nm for the Poly2 layer to reduce the substrate influence. Thus, peak loads of 1000 μN and 1200 μN were chosen for the Au layer, while peak loads of 4000 μN and 5000 μN were chosen for the Poly2 layer. The loading rate was fixed to 25 μN/s and the maximum load was held constant for 5 s to allow time-dependent effects to diminish for Au layer. While the loading rate was fixed to 50 μN/s and the maximum load was held constant for 5 s to allow time-dependent effects to diminish for Poly2 layer. Four nanoindentation tests for each peak load were implemented for both the polysilicon and Au layers. Figure 7 shows the force-displacement curves of the Au layer for different maximum loads. The average Young’s modulus for Poly2 was 161.56 GPa, and the average Young’s modulus for Au was 76.35 GPa. The results obtained by our approaches are in agreement with the values by the nanoindentation test, as shown in Table 5. The obtained residual stresses seem to be reasonable compared with available values given in the literature [26,27,28,29,30,31,32,33] and are in agreement with the PolyMUMPs run data of 105# from the MEMSCAP [34]. This verifies the presented approaches.

In short, the accuracy of our approach is a little far from expectation. Currently, the test accuracy of our approaches is about 10% for Young’s modulus and over 15% for residual stress without practical correction. The inaccuracy due to iterative solutions should be improved. Furthermore, the effects (e.g., residual stress) from the substrate are neglected in our current model. This means that the presented approaches only work on the cases with no or little residual stress in the substrate. Finally, the inter-diffusion between the two layers cannot be considered in our current model. However, this kind of interface layer is inevitable and will affect the accuracy of testing results. Researches to solve these problems should be scheduled as the future work. Of course, if some errors are proved to be systematic deviations, the accuracy may be improved by correction in practical application, just like our MEMS sensors.

## 5. Conclusions

In this paper, a novel measurement method to extract the Young’s moduli and residual stresses for each layer of multilayered films has been presented. Based on the first resonance frequency measurement of the test structure, a theoretical model is proposed and has been firstly verified using the FEA tool. To further verify the proposed method, test structures of two layers are designed, fabricated and tested as an example for simplification. The Young’s moduli and residual stresses for two layer films can be obtained by a set of test structures with different dimensions. The experimental results and available data prove the efficiency of the presented method and this is useful to monitor the MEMS fabrication process.

## Figures and Tables

**Table 1:** Detailed parameters of the test structures with two layers.

Parameters	Length (μm)	Width (μm)		Thickness (μm)	
		Polysilicon	Gold	Polysilicon	Gold
Cantilever beam 1	200	15	9	1.5	0.5
Cantilever beam 2	200	15	5	1.5	0.5
Cantilever beam 3	150	15	9	1.5	0.5
Cantilever beam 4	150	15	5	1.5	0.5
Cantilever beam 5	120	11	9	1.5	0.5
Cantilever beam 6	120	11	5	1.5	0.5
Doubly-clamped beam1	200	15	9	1.5	0.5
Doubly-clamped beam2	200	15	5	1.5	0.5
Doubly-clamped beam 3	120	11	9	1.5	0.5
Doubly-clamped beam 4	120	11	5	1.5	0.5

**Table 2:** Input finite element analysis (FEA) setting parameters of the test structures.

Dimension/Parameter	First Layer	Second Layer
Young’s modulus *E_i_* (GPa)	160	76
Residual stress $\sigma_{i}$ (MPa)	−12	18
	−50	48
	−75	−70
Poisson ratio $\nu_{i}$	0.22	0.42
Density *ρ_i_* (kg/m^3^)	2330	19,300

**Table 3:** Comparisons of the FEA setting Young‘s moduli and residual stresses with the corresponding calculated results from first resonant frequencies simulated by FEA.

Material Parameters	FEA Setting Values	Calculated Results	Relative Error (%)
Young‘s modulus for first layer (GPa)	160	152.36	4.78
Young‘s modulus for second layer (GPa)	76	71.53	5.88
Residual stress for first layer (MPa)	−12	−13.04	8.67
Residual stress for second layer (MPa)	18	16.42	8.78
Young‘s modulus for first layer (GPa)	160	152.77	4.52
Young‘s modulus for second layer (GPa)	76	71.65	5.72
Residual stress for first layer (MPa)	−50	−53.79	7.58
Residual stress for second layer (MPa)	48	44.48	7.33
Young‘s modulus for first layer (GPa)	160	152.08	4.95
Young‘s modulus for second layer (GPa)	76	71.47	5.96
Residual stress for first layer (MPa)	−75	−81.99	9.32
Residual stress for second layer (MPa)	−70	−63.32	9.54

**Table 4:** Test results for the test structures with two layers.

Test Structure	Initially Buckled or Unbuckled	Curvature Radius *R* (μm)	The First Resonance Frequency *f_i_* (kHz)
Cantilever beam 1	buckled	10628.63	39.29
Cantilever beam 2	buckled	14176.24	42.63
Cantilever beam 3	buckled	10765.78	69.87
Cantilever beam 4	buckled	14333.29	75.91
Cantilever beam 5	buckled	10829.78	103.76
Cantilever beam 6	buckled	14467.48	113.67
Doubly-clamped beam1	unbuckled	-	230.12
Doubly-clamped beam2	unbuckled	-	242.00
Doubly-clamped beam 3	unbuckled	-	647.25
Doubly-clamped beam 4	unbuckled	-	700.14

**Table 5:** Test results for various combinations of test structures.

	Results	*E*_1_ (GPa)	*E*_2_ (GPa)	$\sigma_{1}(\mathrm{MPa})$	$\sigma_{2}(\mathrm{MPa})$
Combinations of Test Structures					
Cantilever beam1	Doubly-clamped beam1	151.38	75.34	−13.16	16.27
	Doubly-clamped beam2				
Cantilever beam2	Doubly-clamped beam3	151.38	75.34	−13.11	18.76
	Doubly-clamped beam4				
Cantilever beam3	Doubly-clamped beam1	153.41	72.58	−13.70	17.89
	Doubly-clamped beam2				
Cantilever beam4	Doubly-clamped beam3	153.41	72.58	–14.37	23.18
	Doubly-clamped beam4				
Cantilever beam5	Doubly-clamped beam1	154.93	70.72	–14.08	19.49
	Doubly-clamped beam2				
Cantilever beam6	Doubly-clamped beam3	154.93	70.72	–14.86	23.95
	Doubly-clamped beam4				
Averages by nanoindentation		161.56	76.35	-	-
References		149.3–171.5 [26]	79 [29]	–9 [32]	14.1~35.8 [33]
		160 [27]	74 [30]		
		163 [28]	77.2 [31]		
Values from MEMS CAP [34]		-	-	−10	26

* *E*_1_ is the Young’s modulus of Poly2, *E*_2_ is the Young’s Modulus in the Au layer, $\sigma_{1}$ is the residual stress of Poly2, $\sigma_{2}$ is the residual stress of gold layer.
